# Novel WT1 Target Genes: *IL-2*, *IL-2RB*, and *IL-2RG* Discovered during *WT1* Silencing Using Lentiviral-Based RNAi in Myeloid Leukemia Cells

**DOI:** 10.1155/2020/7851414

**Published:** 2020-10-14

**Authors:** Duangnapa Dejjuy, Chavaboon Dechsukhum, Kovit Pattanapanyasat, Egarit Noulsri, Gregory A Dissen, Wilairat Leeanansaksiri

**Affiliations:** ^1^School of Preclinic, Institute of Science, Suranaree University of Technology, 111 University Avenue, Muang, Nakhon Ratchasima 30000, Thailand; ^2^School of Pathology, Institute of Medicine, Suranaree University of Technology, 111 University Avenue, Muang, Nakhon Ratchasima 30000, Thailand; ^3^Office for Research and Development, Faculty of Medicine Siriraj Hospital, Mahidol University, 2 Wanglang Road, Bangkok Noi, Bangkok 10700, Thailand; ^4^Molecular Virology Core, Oregon National Primate Research Center, Oregon Health and Science University, 505 NW 185th Ave., Beaverton, OR 97006, USA

## Abstract

Wilms' tumor 1 (WT1) is a transcription factor which plays a major role in cell proliferation, differentiation, survival, and apoptosis. WT1 was first identified as a tumor suppressor gene in Wilms' tumor. However, overexpression of *WT1* has been detected in several types of malignancy including some types of leukemia. To investigate the molecular mechanism underlying WT1-mediated leukemogenesis, lentiviral-based siRNA was employed as a tool to suppress *WT1* expression in the myeloid leukemia cell line, K562. Successfully, both WT1 RNA and protein levels were downregulated in the leukemia cells. The silencing of *WT1* resulted in significant growth inhibition in WT1-siRNA-treated cells for 40 ± 7.0%, 44 ± 9.5%, and 88 ± 9.1% at 48, 72, and 96 hours posttransduction as compared with the control cells, respectively. By using apoptosis detection assays (caspase-3/7 activity and Annexin V-FITC/PI assays), *WT1* silencing induced a higher degree of early and late apoptosis in siRNA-treated K562 as compared with the control cells. Interestingly, the expression of survival signaling genes, *IL-2*, *IL-2RB*, and *IL-2RG*, was also suppressed after WT1-siRNA treatment. In addition, the *WT1* silencing also inhibited the S phase of the cell cycle and induced cell death. Our results indicated that WT1 silencing by siRNA can suppress cellular proliferation, induce apoptosis, and reduce S phase fraction of K562 cells. Moreover, transcriptional modulation of *IL-2*, *IL-2RB*, and *IL2-2RG* expression by WT1 was likely involved in this phenotypic change. Overall, this study confirmed the oncogenic role of WT1 in myeloid leukemia and discovered the new target genes of WT1 which are likely involved in WT1-mediated leukemogenesis.

## 1. Introduction

The *Wilms' tumor1* (*WT1*) gene is located on human chromosome 11p13 and is about 50 kb in length and consists of ten exons [1, 2]. It encodes a zinc-finger transcriptional regulatory protein containing four Cys2-His2 Kruppel-like zinc-fingers on their C-terminus. *WT1* was first identified as a candidate tumor susceptibility gene for Wilms' tumor, the most common pediatric renal malignancy [1]. There are at least 36 WT1 isoforms that have been detected in mammalian cells. The diversity of the WT1 structure results from various mechanisms, including alternative mRNA splicing, transcription start sites, translation initiation sites, and RNA editing. The four major WT1 isoforms generated from alternative splicing are WT1(-17AA/-KTS), WT1(+17AA/-KTS), WT1(-17AA/+KTS), and WT1(+17AA/+KTS) also named as WT1 A, B, C, and D isoforms, respectively. These major alternatively spliced WT1 isoforms have been shown to have functional relevance. Each major WT1 isoform is derived from two alternative splicing events; the first event results in the inclusion or exclusion of 17 amino acids encoded by exon 5, which functions as a transactivation domain. The second alternative splicing event results in an inclusion or exclusion of 3 amino acids (KTS: lysine, threonine, and serine) located between the third and fourth zinc-finger domains. The WT1-KTS was shown to function as a DNA binding protein whereas WT1+KTS was shown to possess RNA binding property; therefore, it is likely involved in posttranscriptional regulation [3]. WT1 was also shown to play crucial roles in various physiological functions including cell proliferation, differentiation, survival, and apoptosis [4–6]. The involvement of WT1 in these cellular activities was likely mediated by transcriptional regulation of the WT1 target genes. Intriguingly, the function of WT1 seems to be cellular context-dependent [7]. Indeed, the expression and mutational status of major WT1 interactive proteins including p53 [8, 9] and PAR4 [10] were shown to be able to modify WT1 functions.

The role of WT1 in the carcinogenesis of human malignancies becomes the major area of interest. Although WT1 was first identified as a tumor susceptibility gene in Wilms' tumor, overexpression of the WT1 gene in other types of malignancy suggested its oncogenic role [11]. Aberrant expression of wild-type WT1 was detected in various malignancies, especially breast cancer [12, 13], ovarian cancer [14], hepatocellular carcinoma [15, 16], leukemia [17–19], and neuroepithelial tumor [20]. Moreover, the prognostic value of *WT1* alteration in some cancer was demonstrated. Determination of *WT1* expression by an immunohistochemical method on ovarian carcinoma specimens showed that around 50% of ovarian carcinoma samples possessed a high level of *WT1* expression and the expression level had a negative impact on the survival rate of this cancer [21, 22]. As aberrant expression of WT1 in leukemia is the most consistent finding, several studies addressing the role of WT1 in leukemia have been reported. In AML (acute myelogenous leukemia) patients, the ratio of four major WT1 isoforms A : B : C : D was shown as 17 : 23 : 24 : 31% while the ratio of these isoforms was 10 : 16 : 7 : 39% in normal CD34^+^ cells. This result indicated that each WT1 isoform has a different impact on leukemogenesis [23]. The physiological relevance of the alteration in the ratio of major WT1 isoforms was recapitulated. The preferential expression of WT1 isoforms A, B, and C was detected in AML patients [24]. In addition, prognostic value *WT1* expression was demonstrated in CML (chronic myelogenous leukemia), in which a high level of WT1 expression is detected in CML patients with relapse while remaining low in the patients with complete remission [18, 25].

The involvement of other specific WT1 isoforms in carcinogenesis has been also investigated. One of the truncated WT1 isoforms was also detected in some types of cancer such as prostate cancer cell line, K562 (myeloid leukemia cell line), MCF-7 (breast cancer cell line), and acute leukemic blood samples [26]. In addition, overexpression of full-length *WT1* and truncated *WT1* was observed in approximately 94.5% and 19% in AML patients, respectively [27]. Recently, another novel truncated WT1 isoform called Ex4a (+) WT1 isoform was observed in myeloid leukemia and solid tumor cells [28]. The overexpression of Ex4a (+) WT1 suppressed transcription of *Bcl-xl* gene and induced mitochondrial damage and apoptosis.

Functional studies to scrutinize the molecular pathway underlying WT1-mediated leukemogenesis have been reported. Downregulation of *WT1* gene resulted in reduced cell proliferation with G0/G1 arrest of the cell cycle in myeloid leukemia cells [29]. Moreover, suppression of WT1 by curcumin inhibited growth and induced G2/M arrest in myeloid leukemia cells [30]. Furthermore, *WT1* silencing by shRNA resulted in an increased proportion of cells in the G0/G1 phase and a reduced proportion of cells in the S phase in these leukemia cells [30]. Although most of the studies supported the oncogenic role of WT1 in leukemia, some conflicting data were reported. Overexpression of *WT1* induced differentiation of myeloblastic leukemia M1 cells [31]. Moreover, a low level of W1 protein expression was reported in T-ALL cell lines and primary T-ALL cells from the patients. In addition, induced WT1 expression in this T-ALL cell resulted in upregulation of CD95L expression and enhancement of CD95L-mediated cell death [32]. Recently, WT1 was shown to interact with TET2, and this interaction is critical for growth inhibition of leukemia cells [33]. These discrepancy results addressed the cellular context-dependent role of WT1. Besides the leukemia model, effects of *WT1* silencing on the malignant phenotype of other tumor cells supported the oncogenic role of WT1. *WT1* silencing inhibited the MG-63 cell line by cell cycle arrest and apoptosis activation [34]. Moreover, *WT1* knockdown inhibited proliferation of the malignant peripheral nerve sheath tumor sNF96.2 cell line [35].

In this study, we designed a new WT1-siRNA and delivered to the K562 leukemia cells by using the pPRIME-CMV-GFP-FFs lentiviral vector system. This WT1-siRNA-lentiviral system will be employed to investigate the role of WT1 in leukemogenesis as well as the potential underlying molecular mechanism.

## 2. Materials and Methods

### 2.1. Cell Culture

The human myeloid leukemia cell line (K562) was cultured in complete Roswell Park Memorial Institute Medium (cRPMI 1640) (Gibco). Packaging cells (293T/17) were cultured in complete Dulbecco's modified Eagle's medium (cDMEM) (Gibco). Culture media were supplemented with 10% fetal bovine serum (FBS) (Hyclone), penicillin (100 U/ml), and streptomycin (100 *μ*g/ml). Cells were maintained in a humidified incubator at 37°C and 5% CO_2_. cDMEM media without antibiotics were used for further lentiviral production.

### 2.2. Lentiviral Production, Viral Titer Determination, Transduction, and GFP^+^ Cell Sorting

The new sequences of WT1-siRNA and scramble siRNA (control sequence) were designed by siRNA target designer software (Promega) and cloned into pPRIME-CMV-GFP-FFs lentiviral vector, which was a gift from Prof. Dr. Gregory Dissen (Oregon National Primate Research Center, USA). The lentiviral vector was further produced by using the calcium chloride precipitation method. Briefly, three packaging plasmids (6.5 *μ*g of pLP1, 2.5 *μ*g of pLP2, and 3.5 *μ*g of pLPv) and 10 *μ*g of pPRIME-CMV-GFP-FFs-C-siRNA vector (control vector) or pPRIME-CMV-GFP-FFs-WT1-siRNA vector were transfected into 80% confluence of 293T/17 packaging cells in 100 mm cell culture dish (CellStar). Lentiviral supernatant was harvested and filtrated through 0.45 *μ*m filter and then concentrated by ultracentrifugation at 28,000 rpm at 4°C for 2 hours. For the transduction procedure, K562 cells were cultured in cRPMI media overnight prior to transduction. Two million K562 cells were added into 10x viral supernatant. To increase viral transduction efficiency, Polybrene (Sigma) was added to the cell suspension to 8 *μ*g/ml final concentration and the cell suspension was then subjected to centrifugation at 1,800 rpm, 25°C for 45 minutes. The targeted cells were further incubated for 12 hours at 37°C, 5% CO_2_. After transduction, K562 cells were incubated in cRPMI medium for 48 hours at 37°C in 5% CO_2_. GFP^+^ cells were sorted by FACSVantage (BD Biosciences). The GFP^+^-K562 cells were maintained in cRPMI 1640 at 37°C, 5% CO_2_, and 90% humidity until used.

### 2.3. Cell Proliferation Assay

WT1-siRNA-GFP^+^-K562 cells and C-siRNA-GFP^+^-K562 cells were plated at a density of 10,000 cells/100 *μ*l/well in a 96-well plate. Cell proliferation was analyzed at various time points of 0, 3, 6, 12, 24, 48, 72, and 96 hours posttransduction by adding 20 *μ*l/well of the CellTiter 96 Aqueous One solution reagent (Promega) prior to further incubation for 2 hours at 37°C, 5% CO_2_. After that, 25 *μ*l of 10% SDS was added to each well and subjected to the measurement of the absorbance at 490/620 nm using an ELISA plate reader.

### 2.4. Apoptosis Assay Using Apo-One Homogeneous Caspase-3/7 Reagent

K562-WT1-siRNA-GFP^+^ cells and K562-C-siRNA-GFP^+^ cells were plated at a density of 10,000 cells/100 *μ*l/well in 96-well plates. One hundred microliters of Apo-One Homogeneous Caspase-3/7 reagent (Promega) was added into each well at specific time points of 0, 3, 6, 12, 24, 48, 72, and 96 hours posttransduction. The mixtures were further incubated at room temperature in the dark environment for 4 hours and subjected to absorbance evaluation at 499_EX_/521_EM_ nm by using a fluorescent spectrometer (RI Technologies). The absorbance value was represented as caspase-3/7 enzyme activities which are directly correlated with the apoptosis induction.

### 2.5. Apoptosis Assay Using Flow Cytometry

K562-WT1-siRNA-GFP^+^ cells and K562-C-siRNA-GFP^+^ cells were stained with Annexin V-FITC and propidium iodide (PI) using an Annexin V-FITC/PI Apoptosis detection kit (BD Pharmingen) according to manufacturer instruction. Briefly, the cells were washed twice with PBS. The pellet of cells was resuspended with 100 *μ*l of 1x buffer solution provided in the reagent kit. Five microliters of Annexin V-FITC and PI was added into the cell suspension and incubated at room temperature in the dark environment for 15 minutes. At the end of incubation, 400 *μ*l of 1x buffer solution was added into the cell mixture and the cells were then subjected to flow cytometry analysis by using FACSCalibur (BD Biosciences) and CellQuest Pro software.

### 2.6. Reverse Transcriptase Polymerase Chain Reaction (RT-PCR)

K562-WT1-siRNA-GFP^+^ cells and K562-C-siRNA-GFP^+^ cells were harvested for total RNA extraction using a total RNA mini kit (Geneaid), and the obtained total RNA solutions were treated with RNase inhibitor (Invitrogen) according to the manufacturer protocol. The cDNA was generated by a RevertAid™ First Strand cDNA synthesis kit (Fermentas) according to manufacturer procedures. The newly synthesized cDNA was amplified by PCR. Each PCR reaction was carried on by adding 10 *μ*l cDNA template solution into the PCR mixture containing 1x PCR buffer, 0.2 mM mixed dNTPs, 2 mM MgCl_2_, 0.2 pmole reverse primer and forward primer (Table 1), and 1 unit of Taq DNA polymerase. The amplification cycle was done as follows: 95°C for 5 minutes for initial denaturation; then, 40 cycles of amplification step were done at 95°C denaturation for 1 minute, appropriated temperature for 30 seconds for annealing step (Table 1), 72°C for 45 seconds for extension step, and then 72°C for 5 minutes for the final extension. The PCR products were examined by 1.5% agarose gel electrophoresis, and PCR fragments were visualized by GelDoc (Bio-Rad) based on ethidium bromide staining.

### 2.7. Western Blot Analysis

K562-WT1-siRNA-GFP^+^ cells and K562-C-siRNA-GFP^+^ cells were lysed by the CelLytic M reagent (Sigma) for 15 minutes. Protein lysate was centrifuged at 12,000 rpm for 15 minutes, and the protein concentrations were determined using the Bradford protein assay (Sigma). Twenty micrograms of protein was separated on SDS-PAGE and then transferred to a PVDF membrane (Whatman) using blotting buffer for 1 hour. Then, the membrane was blocked for 1 hour and probed with specific primary antibodies (1 : 100 of polyclonal anti-WT1 antibody (Santa Cruz, C19) or 1 : 1000 of polyclonal anti-actin antibody (Santa Cruz, H196) or 1 : 1000 of anti-caspase-7 antibody (Sigma, C7724) in 1% skim milk at room temperature for 2 hours. Consequently, the membrane was detected with the immunocomplexes using horseradish peroxidase conjugated with either an appropriated dilution of goat anti-mouse or goat anti-rabbit secondary antibodies (Santa Cruz). The immunocomplex was detected with SuperSignal Pico Chemiluminescent Substrate for 5 minutes followed by film exposure.

### 2.8. Cell Cycle Analysis

To further investigate the mechanism by which WT1-siRNA induce K562 cell death, K562 was transduced with WT1-siRNA or C-siRNA. After GFP expression, total GFP-positive cells were sorted. K562-WT1-siRNA-GFP^+^ and K562-C-siRNA-GFP^+^ cells were corrected for cell cycle determination at 24 and 72 hours posttransduction. Briefly, total 1 × 10^5^ cells were packed and washed twice with PBS. Consequently, cells were suspended with 500 *μ*l of PBS; then, cell suspension was dropped into 4 ml of cold 70% ethanol with gentle shaking on the vortex mixer for cell clumping protection. Cells were incubated in 70% ethanol on ice for more than 1 hour. After that, fixed cells were packed by centrifugation at 2,000 rpm, 4°C for 5 minutes. Supernatant was carefully discarded to prevent the loss of cell pellets. Cells were washed with 5 ml PBS and centrifuged at 2,000 rpm, 4°C for 5 minutes, and cells were stained with 1 ml propidium iodide master mix (freshly prepared: 40 *μ*l of 40 *μ*g/ml PI and 3.5 *μ*l of 100 *μ*g/ml RNase A with the volume adjusted to 1 ml with ddH_2_O) for 15 minutes at 37°C in water bath. Cells were analyzed by flow cytometry using a DNA setup protocol (Becton Dickinson).

### 2.9. Statistics

All data are expressed as the mean ± SD. Differences between the treated and control cells were analyzed by a *t*-test. A probability of *p* < 0.05 was considered to be significant.

## 3. Results

### 3.1. WT1-siRNA Lentiviral Transduction Downregulated Endogenous WT1 Expression

**Figure 1:**
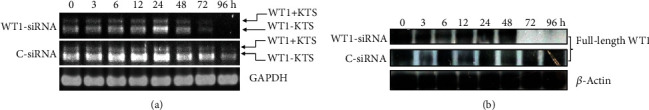
WT1-siRNA lentiviral transduction suppressed endogenous WT1 mRNA expression level at 48 hours posttransduction. RT-PCR analysis of WT1 was performed. Two specific PCR bands representing WT1(+KTS) and WT1(-KTS) transcripts were downregulated (a). WT1 protein was also reduced by WT1-siRNA lentiviral transduction. Intriguingly, no detectable full-length WT protein is observed at 72 and 96 hours posttransduction in *WT1* silenced cells.

After lentiviral transduction was performed, green fluorescent protein (GFP) expressed on the cells was used as a tracking marker for the successfully transduced cells. Total GFP^+^ cells were collected for WT1 expression determination along with cellular and molecular analyses. The endogenous WT1 gene expression was downregulated after 48 hours posttransduction with WT1-siRNA lentiviral vector. Our result demonstrated the decreased level of 215 bp and 225 bp WT1 RT-PCR products which represented WT1(-17AA) and WT1(+17AA) mRNA levels, respectively, in WT1-siRNA lentiviral vector-transduced cells as compared to the control cells. The result indicated that specific WT1-siRNA used in our experiments can induce downregulation of endogenous WT1 mRNA in K562 cells (Figure 1(a)). Consistent with mRNA results, WT1-siRNA lentiviral vector transduction also induced WT1 protein suppression. The samples of K562-WT1-siRNA-GFP^+^ cells and K562-C-siRNA-GFP^+^ cells were collected, and protein expression analysis was performed by western blot analysis. The result demonstrated that the full-length WT1 was downregulated in the WT1-siRNA lentiviral vector-transduced cells at 72 and 96 hours posttransduction (Figure 1(b)). Our result showed the successful knockdown of WT1 expression at both mRNA and protein levels by using WT1-siRNA introduced into the cells by the lentiviral system.

### 3.2. WT1-siRNA Inhibited Cell Growth of K562 Cells

**Figure 2:**
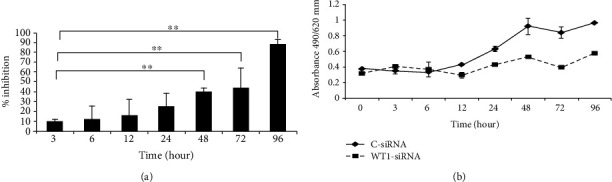
WT1 silencing (WT1-siRNA) induced growth inhibition of K562 cells. Inhibitory effect of WT1-siRNA on K562 cell growth was calculated by subtraction of viable cells at specific time with a viable number at 0 hours of the experiment and was presented as the percentage of growth inhibition (a). WT1 silencing induced the inhibition of cellular proliferation of K562 cells detected by using the MTT assay compared with the control cells (C-siRNA) (b). Data represented the average value of three independent experiments. ^∗^*p* < 0.05; ^∗∗^*p* < 0.01 by paired samples *t*-test.

The GFP^+^ K562 cells were further evaluated for the physiological effects of WT1 gene silencing by a growth inhibition assay. Our results showed that WT1-siRNA significantly inhibited the survival of K562-WT1-siRNA-GFP^+^ cells as 60 ± 6%, 56 ± 14%, and 12 ± 3% cell survival are observed at 48, 72, and 96 hours posttransduction, respectively. When compared with K562-C-siRNA-GFP^+^ cells, the degree of growth inhibition in WT1 silencing K562 cells was 40 ± 7%, 44 ± 21%, and 88 ± 9% at 48, 72, and 96 hours posttransduction, respectively (Figure 2(a)). Indeed, WT1-siRNA suppressed the proliferation of K562 cells whereas the growth of K562-C-siRNA-GFP^+^ cells was increased (Figure 2(b)). These results indicated that the lentiviral vector is a powerful tool for siRNA delivery into the myeloid leukemia cells. Moreover, the lentiviral vector containing the GFP reporter gene allowed the easy way to measure the efficiency of viral transduction and for monitoring of molecular and cellular alterations of the interested cells. By using this strategy, the growth inhibitory effect of WT1-siRNA sequence on K562 can be clearly demonstrated. These results suggested that WT1 is an important factor for cell survival and to support leukemia cell proliferation. Our finding confirmed that WT1 plays an oncogenic role in the leukemia cells.

### 3.3. Apoptosis Induction by WT1-siRNA

**Figure 3:**
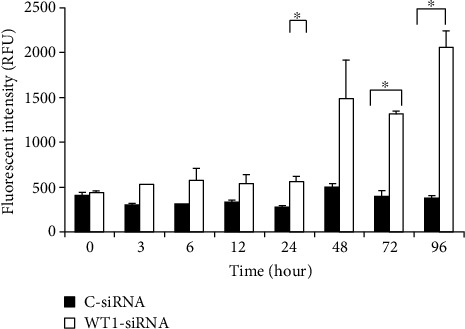
WT1 silencing (WT1-siRNA) induced apoptosis via activation of caspase-3/7 enzyme activity of K562 cell (K562-WT1-siRNA-GFP^+^ cells) at the specific time points compared with the C-siRNA-GFP^+^ control cells (C-siRNA). Analysis of caspase-3/7 enzyme activities was performed by using the Apo-One Homogeneous Caspase-3/7 apoptosis kit (Promega).

**Figure 4:**
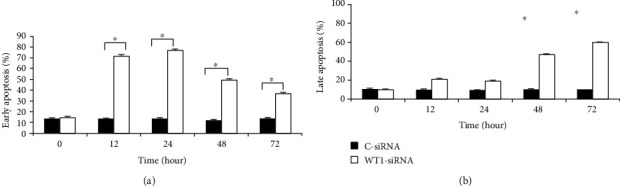
WT1 silencing (WT1-siRNA) induced apoptosis of K562 cells. The K562-WT1-siRNA-GFP^+^ cells (WT1-siRNA) and C-siRNA-GFP^+^ (C-siRNA) control cells were harvested at 0, 3, 6, 12, 24, 48, 72, and 96 hours posttransduction. Flow cytometry analysis was performed based on Annexin V-FITC/PI staining for apoptotic cell determination. Early apoptotic (a) and late apoptotic (b) cells were calculated based on the percentage of Annexin V^+^ and Annexin V^+^/PI^+^ cell population, respectively. The assay was performed in triplicate (^∗^*p* < 0.05 by paired samples *t*-test).

We further pursued an apoptotic assay to evaluate the possible role of WT1 in the apoptosis regulation of leukemia cells. In this study, apoptotic cells were determined based on the increasing levels of caspase-3 and caspase-7 apoptotic enzyme activities and also by detection of cell membrane change during the apoptotic process by Annexin V-FITC/PI staining assay. The result showed that WT1-siRNA effectively induced K562 cell apoptotic cell death. Moreover, apoptotic enzyme activity was also evaluated based on a fluorescence-based assay. Caspase-3/7 activity in WT1-siRNA-treated cells was measured as 1,487 ± 425, 1,319 ± 31, and 2,051 ± 189 RFU at 48, 72, and 96 hours, respectively (Figure 3). The result showed that caspase-3/7 activity was significantly higher in WT1-siRNA-treated cells as compared with the control cells, in which caspase-3/7 activity in WT1-siRNA-treated cells was 3-folds and 4-folds higher than the control cells at 48 hours and 96 hours posttransduction, respectively. These results suggested that WT1-siRNA could induce apoptosis via the activation of apoptotic enzymes: caspase-3 and/or caspase-7. In addition, we confirmed the effects of WT1-siRNA on apoptotic induction in K562 cells by another assay. In this assay, the early cell apoptosis was assessed by measurement of the quantity of Annexin^+^ cells while late apoptosis was assessed by measurement of the quantity of double-positive Annexin and PI. These results showed that early apoptotic cells observed in WT1-siRNA-treated cells were 71 ± 1%, 77 ± 0.49%, 50 ± 0.9%, and 36 ± 1.2% at 12, 24, 48, and 72 hours posttransduction, respectively (Figure 4(a)). In addition, late apoptosis was demonstrated to be 48 ± 0.93% and 60 ± 1.23% at 48 and 72 hours posttransduction, respectively (Figure 4(b)). This result implied that apoptotic induction contributed to growth inhibitory effect of WT1 gene silencing in leukemia cells.

### 3.4. WT1-siRNA Inhibited IL-2 Survival Signaling Genes of Transduced K562 Cells

**Figure 5:**
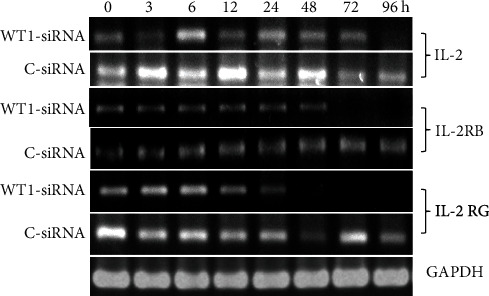
WT1 silencing (WT1-siRNA) downregulated the expression of *IL-2*, *IL-2RB*, and *IL-2RG* compared with the control cells (C-siRNA). RT-PCR analysis of IL-2, IL-2RB, and IL-2RG mRNA was performed on RNA isolated from K562-WT1-siRNA-GFP^+^ or K562 cells after WT1-siRNA lentiviral vector transduction (WT1-siRNA) and K562-C-siRNA-GFP^+^ control cells (C-siRNA). GAPDH mRNA detection was used as an internal control.

We next investigated whether the WT1 knockdown in K562 cells has any impact on other gene expressions, especially those involved in the growth signaling pathway in myeloid cells. By this approach, the molecular pathway underlying WT1-mediated leukemogenesis could be elucidated. Expression of interleukin-2 (IL-2) gene, encoding a key cytokine involved in leukemia cell survival, was evaluated by RT-PCR (Figure 5). This result showed that the IL-2 mRNA expression level was decreased in WT1-siRNA-transduced cells at 96 hours posttransduction as compared with the control cells. These results suggested that inhibition of the IL-2 signaling pathway likely contributes to growth inhibitory effect of WT1 gene silencing on K562 leukemia cells. IL-2 receptor consists of three receptor subunits including alpha subunit (IL-2RA), beta subunit (IL-2RB), and gamma subunit (IL-2RG). Heterotrimerization of all subunits (*α*, *β*, and *γ*) is classified as a high-affinity receptor for IL-2 binding and therefore mediated the strongest signal into the cells. However, heterodimer of IL-2RA and IL-2RB show intermediate affinity for IL-2 binding. In this study, IL-2 receptor subunit gene expression at the RNA level was evaluated. The results showed that the expression of IL-2RG and IL-2RB mRNAs was decreased until reaching an undetectable level at 48 and 72 hours posttransduction, respectively, in K562-WT1-siRNA-GFP^+^ (WT1-siRNA) leukemia cells. The result suggested that WT1 gene silencing suppresses the function of IL-2RB and IL-2RG at the transcriptional level. These results indicated that modulation of IL-2 and IL-2 receptor subunit (*β* and *γ*) gene expression involves in the growth inhibition mechanism induced by WT1 gene silencing.

### 3.5. WT1 Silencing Decreased S Phase Fraction

**Figure 6:**
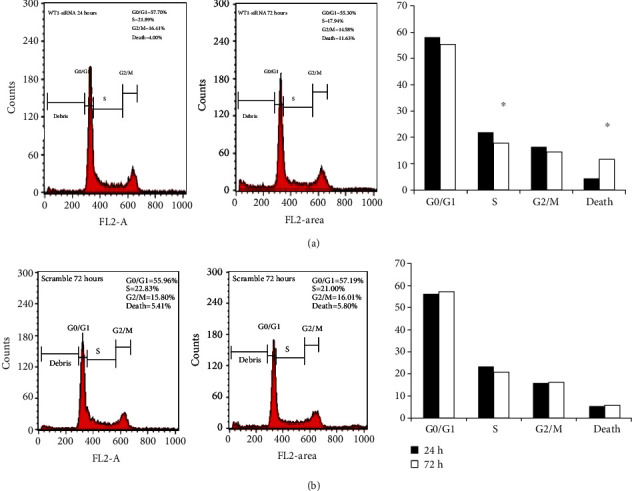
Effect of WT1 silencing on cell cycle progression of K562 cells. Determination of cell cycle distribution of K562-WT1-siRNA-GFP^+^ compared with K562-C-siRNA-GFP^+^ control cells at 24 and 72 hours posttransduction by flow cytometry. The distribution of cells in the G0/G1, S, G2/M, and death cells was calculated and labeled. The percentage of each phase was plotted, C-siRNA population (Panel A) and WT1-siRNA-treated cell population (Panel B). The assay was performed in triplicate. Statistical significance was determined using Student's *t*-test; *p* < 0.01.

To evaluate the effect of WT1 gene silencing on cell cycle progression, transduced cells were analyzed by flow cytometry. The result showed that WT1 gene silencing induced cell death up to 11.6% in WT1-siRNA-treated cells at 72 hours posttransduction that was significantly higher than the control cells (5.80% cell death). Moreover, we found a significant reduction of cells in the S phase (S phase fraction) in WT1-siRNA-treated cells from 21.8% at 24 hours posttransduction to 17.9% at 72 hours posttransduction (Figure 6). This result suggested that WT1-siRNA can inhibit cell cycle progression by blocking the cell from entering the S phase.

## 4. Discussion

In this study, we reported the accomplishment in the production of lentiviral-based RNAi which effectively inhibited WT1 expression in acute myeloid leukemia cells, K562. Both WT1 mRNA and WT1 protein levels were significantly reduced in the K562-WT1-siRNA-GFP^+^ population as compared with the control group along the whole observation period (Figures 1(a) and 1(b)).

However, we observed that WT1 protein was also decreased in the control cells at 96 hours posttransduction. Based on the evidences in the literature, this finding is likely due to the physiologic change of the WT1 expression level that was shown to be associated with the proliferation rate of the cells. It was demonstrated that the *WT1* expression level is associated with the cell proliferation rate and cell cycle phase of the K562 myeloid leukemia cells (the higher proliferation rate, the higher WT1 expression while the lower proliferation rate, the lower WT1 expression) [29, 30]. According to this scenario, the control cells which are not subjected to *WT1* silencing grew very fast and reached high cell density leading to slowing down of the proliferation rate of the cells at 96 hours posttransduction. Therefore, declination of the proliferation rate by high cell density in culture can lead to a decrease in WT1 expression in these cells which may account for the slight decrease in the WT1 mRNA level at 96 hours posttransduction in the control cells (Figure 1(a)).

Functional and molecular studies to identify the effect of *WT1* silencing on key cellular functions in this work showed that downregulation of *WT1* expression resulted in dramatic growth inhibition, enhanced apoptosis, and S phase progression inhibition in K562 leukemia cells. These results can be further developed for clinical application as a WT1-targeted therapy in this type of leukemia. However, further studies in other myeloid leukemia cells and in clinical samples would be worthwhile. Our results are in line with some previous reports showing that downregulation of WT1 by using antisense oligonucleotides led to inhibition of cellular proliferation of K562 and fresh leukemia cells from AML and CML patients [39]. Moreover, it has been shown that in cell culture condition, the reduction in the serum level or the decreased pH in culture media led to growth inhibition of CML and AML primary cells obtained from leukemia patients. Interestingly, this growth suppressive effect was shown to be mediated by WT1 downregulation [29]. In addition, using the anti-WT1 strategy combined with curcumin plant extract resulted in reduced K562 cell proliferation by dose- and time-dependent manners via the inhibition of the protein kinase C (PKC) pathway [40]. Furthermore, overexpression of WT1(+17AA/+KTS) and WT1(-17AA/-KTS) isoforms resulted in delayed differentiation of K562 cells induced by 12-o-tetradecanoyl phorbol 13-acetate [41]. However, the conflicting data was also reported in that WT1(+KTS) overexpression can promote monocytic differentiation in murine promyelocytic leukemia M1 [42].

With regard to apoptosis, specific silencing of WT1(+17AA) by siRNA induced apoptosis via activation of caspase-3 and caspase-9 in myeloid leukemia cells [43]. In contrast, a previous report showed that WT1(-17AA,-KTS) isoform could repress the promoter of *BCL-2* therefore functioning as a proapoptotic molecule in Hela cells, which supported the role of WT1 as a tumor suppressor gene [7]. These controversial findings highlighted the functional differences among WT1 isoforms. With respect to cell cycle regulation, downregulation of WT1 expression in this work resulted in cell cycle arrest of K562 cells. This result is supported by the previous study showing that the inhibition of WT1 expression caused G2/M arrest in leukemic cells [44]. Additionally, downregulation of WT1 resulted in reduced S phase progression on K562 cells and accumulated cells in the G1 phase [29]. In contrast to human cells, in overexpression of WT1 in murine myeloblastic leukemia (M1) cell line, the cells exhibited G1 arrest and apoptosis [45]. Therefore, the role of WT1 in cell cycle regulation is likely dependent on the cellular context [44].

Based on the literature review, this study is the first report to show that WT1 can regulate the RNA expression of *IL-2*, *IL-2RB*, and *IL-2RG* in the myeloid leukemia cells. This data provide new insight into the molecular mechanism underlying the WT1-mediated leukemic transformation process. Based on the evidences in literatures, WT1 has not been shown to transcriptionally regulate the expression of these genes. The only related EWS-WT1 fusion protein was found to be able to transcriptionally control *IL-2RB* [38]. As WT1 has been shown to possess different roles largely depending on the cellular context, the understanding of the molecular network of signaling pathways initiated by WT1 in each specific type of cancer would be beneficial to design tailored molecular-based therapy suitable for any specific cancer. However, further functional studies are required to confirm this observation.

In fact, IL-2 is a T cell-derived cytokine which plays critical roles in cellular proliferation, differentiation, and immune activation. In addition, IL-2 plays a critical role in growth and signaling pathway of hematopoietic cells, especially T cell; the interplay between WT1 and IL-2/IL-2 receptors possibly contributes significantly to the WT1-mediated leukemogenesis [46]. The IL-2 signaling pathway is initiated by the interaction with IL-2 receptors consisting of alpha-beta-gamma subunits (ABG). This heterotrimeric complex is the high affinity receptor and produces the strongest downstream signal. However, heterodimer of alpha-beta subunits (AB) or beta-gamma subunits (BG) can induce the IL-2 signaling pathway with lesser activity. The heterodimerization between IL-2RB and IL-2RG can mediate intracellular signals for T cell proliferation [47, 48]. In addition, the cytoplasmic domain of IL-2RG induces Jak/Stat activation via the phosphorylation of Jak3 molecule that further activates Stat phosphorylation. The phosphorylated-Stat molecules are then homodimerized and moves into the nucleus to activate target gene transcription and subsequently cytokine production [49]. However, the role of the IL-2 signaling pathway in myeloid leukemia cells is largely unclear. Moreover, the research data elucidating the molecular mechanism underlying IL-2-mediated AML development is limited. The study for the role of IL-2 as a possible prognostic indicator for AML was recently published [50]. According to this research work [50], the IL-2RA mRNA level as detected by real-time RT-PCR is significantly correlated with poor outcomes of the patients in terms of relapse-free survival and overall survival rate regardless of conventional prognostic subgroups including favorable, intermediate, and poor-risk AML. Moreover, there was a correlation between IL-2RA mRNA expression and mRNA expression of other genes implicated in the AML oncogenic process including *FLT3*, *ID1* which are the targets of oncogene tyrosine kinase. Furthermore, the association between IL-2RA mRNA and mRNA expression of *EGR* and *CDKN1B*, a stem cell-liked feature signature, was also demonstrated. However, the refined molecular pathway underlying the interplay between IL-2RA and these proteins remained to be elucidated. Due to the fact that WT1 was able to regulate both IL-2 and its receptor expression simultaneously, the influence of WT1 expression on the IL-2 signaling pathway is likely to be potentiated. Further functional studies are required to confirm this scenario.

## 5. Conclusions

This work demonstrated the achievement of using a newly designed siRNA against WT1 gene to inhibit endogenous WT1 expression in K562 leukemia cells. A lentiviral-based system was shown to be a powerful siRNA delivery method. The effect of WT1 silencing on cell proliferation and apoptosis was monitored, and the molecular pathway underlying these effects was also investigated. The results show the new insight into WT1-mediated leukemogenesis. WT1 gene silencing in these leukemia cells resulted in growth inhibition and enhanced apoptosis and decreased S phase fraction. The attempt to identify downstream molecules involved in WT1-mediated leukemic transformation showed the novel finding. WT1 silencing led to downregulation of the RNA expression of *IL-2*, *IL-2RB*, and *IL-2RG*. Our study supported the oncogenic role of WT1 in myeloid leukemia, and the highlight of this work is the discovery of new WT1 target genes, *IL-2*, *IL-2RB*, and *IL-2RG*, which are likely involved in WT1-mediated leukemogenesis in acute myelogenous leukemia. In conclusion, our study will not only add new members to the plethora of WT1 target genes but also provide new insight to the signaling pathway underlying the WT1-mediated leukemic transformation process.

## Figures and Tables

**Table 1:** The primer sequences and PRC conditions used in RT-PCR analysis.

Genes	Primer sequences	Annealing temperature (°C)	Product size (bp)	References
*WT1*	R: 5′-TCAAAGCGCCAGCTGGAGTTT-3′ F: 5′-AGACATACAGGTGTGAAACC-3′	51	225 (+17AA) 215 (-17AA)	[36]
*IL2*	R: 5′-TGGGAAGCACTTAATTATCAAGTC-3′ F: 5′-CGTAATAGTTCTGGAACTAAAGGG-3′	60	150	[37]
*IL-2RB*	R: 5′-CGGTGTTCCTGCAGTTG-3′ F: 5′-CAGTATGAGTTTCAGGTGCG-3′	50	205	[37]
*IL-2RG*	R: 5′-CCAACAGAGATAACCACGG-3′ F: 5′-CGCTACACGTTTCGTGTTC-3′	60	152	[37]
*GAPDH*	R: 5′GTACTCAGCGGCCAGCATCG-3′ F: 5′-AGCCACATCGCTCAGACACC-3′	60	310	[38]

## Data Availability

All of the in vitro cell culture experimental data and molecular analysis data used to support the findings of this study are included within the article.
